# Veterinary Guidelines for Electrochemotherapy of Superficial Tumors

**DOI:** 10.3389/fvets.2022.868989

**Published:** 2022-07-27

**Authors:** Matías Tellado, Lluis M. Mir, Felipe Maglietti

**Affiliations:** ^1^VetOncologia, Buenos Aires, Argentina; ^2^Université Paris-Saclay, CNRS, Gustave Roussy, Metabolic and Systemic Aspects of Oncogenesis (METSY), Île-de-France, France; ^3^Instituto Universitario del Hospital Italiano-CONICET, Buenos Aires, Argentina

**Keywords:** electroporation, cancer, oncology, pets, dogs, cats, horses, procedures

## Abstract

Electrochemotherapy (ECT) consists in the application of electric pulses to increase chemotherapeutic drug intake (bleomycin, cisplatin, or calcium) into the tumor cells. It has become a very valuable treatment option in veterinary oncology. It is an effective and safe treatment modality, which is not only beneficial as a palliative treatment, but also for a curative approach. Performing the treatment adequately will ensure the best results possible, in the minimum number of sessions, and reduce complications. Usually, only one session is enough to achieve excellent results, but the treatment can be repeated. Several sessions can be necessary in the case of incompletely treated or very extended lesions, as well as in the occurrence of new lesions. ECT is effective for superficial or oral tumors of any histology that are accessible to the electrodes. Intravenous bleomycin is the preferred drug and route of administration, leaving other ways of administration and drugs for selected cases. The guidelines presented here are destined to veterinarians who want to develop their understanding of the basis of ECT and wish to perform it adequately and effectively. In this paper, we also discuss common problems and how to solve them, and we include practical tips to improve the treatment results based on common questions and mistakes of beginner users.

## Introduction

### Electrochemotherapy: Concept, Field of Application, and Advantages

Electrochemotherapy (ECT) is a well-established treatment modality that has been performed in veterinary medicine since 1997 in Europe (1) and since 2008 in Latin America. Its use in human medicine began with a clinical trial published in 1991 (2, 3) and became a standard therapy in 2006 when the standard operating procedures for ECT in human patients were published, and the appropriate equipment was made available (4).

In veterinary medicine, ECT as a standard of care is now available in many countries across the globe. The use of ECT had a steep growth after veterinary electroporators were produced and training courses became available, for example in Slovenia, Brazil and Argentina. The impact solely in Latin America is revealed by the fact that annual meetings in Brazil and Argentina gather users from around the world to share their knowledge and experience. Despite a relatively low number of publications until now, more than 20,000 patients had been treated in more than 120 centers just in Latin America (information gathered from the last users meeting held in Brazil in 2019).

In its beginnings, ECT was used for the palliative treatment of cutaneous and subcutaneous tumors of any histology. But nowadays, it can be used as a first-line treatment, alone or in combination with other therapies. It has been used to treat tumors in different anatomical regions, aided by the electrodes specially designed for veterinary and human medicine (5–11).

ECT consists in the transient and reversible permeabilization of cells through the application of an electric field. This increases the cellular uptake of certain molecules, augmenting their cytotoxic and anticancer efficacy by up to 1,000-fold (12). Bleomycin, cisplatin, or calcium are the drugs proven to be effective, with a clear advantage for bleomycin among them (13, 14). The reasons behind this advantage are that bleomycin selectively kills replicating cells, preserving healthy non-replicating tissues, induces an immune system response, and it can be administered intravenously, providing an adequate dose availability in the treatment area (13, 15).

As the drug is introduced into the cell by a physical phenomenon, penetration is not cell-type dependent. Tumors of any histology can be treated with very good results, with an objective response rate of around 70–100%. This high response rate can be observed in dogs, cats, horses, and of course in human patients as well (11, 16, 17). In particular, in malignant melanoma, the objective response rate in early stages is around 90% (9), in squamous cell carcinomas is around 80% (18), in sarcoids is around 97% (11), and in mast cell tumors of less than 2 cm^3^ is around 100% (19).

The application of the electroporation pulses also induces a *vascular-lock* phenomenon that produces the instantaneous interruption of the blood flow in the treated area. This provides additional benefits to the treatment, i.e., entrapment of the drug inside the tumor, tumor starvation, and immediate hemostasis in the treated area (20, 21).

The immune response plays a crucial role in the efficacy of the treatment, as it was demonstrated in immunocompromised mice that showed a remarkably lower response to the treatment (13, 22, 23). Also, in veterinary medicine, the role of the immune system is very important as it increases treatment effectiveness, and can even give rise to abscopal effects in some particular cases (24).

All in all, a very good response can be obtained in one or two sessions for most cases, depending on the size and location of the tumor. It is also important to recall that the therapy can be combined with other treatment modalities increasing their effectiveness, for example in combination with debulking surgery, chemo, or radiotherapy (25). Finally, ECT can be applied when no other treatment options are available, still with good results, making ECT a very attractive tool for the veterinary oncologist (26).

In precision medicine, treatments are selected and personalized regarding patients' characteristics, genetics, and environmental factors, for increasing their effectiveness and reducing side effects (27). Precision medicine relies on the biological and chemical particularities of the patient and its disease on the one hand, and on the biological and chemical effects of the treatment on the other hand. ECT is an original contribution to precision medicine, as it is a physical approach. It is important to note that all the classical physical approaches up to now (including radiotherapy, brachytherapy, cryotherapy, hyperthermia, high frequency focused ultrasound, and even surgery as a ≪ mechanical ≫ approach) are ablative methods, meaning that they do not selectively discriminate between cancerous and normal cells. On the contrary ECT is not an ablative approach. As will be discussed in the next sections, the exquisite combination of electropermeabilization and a non-permeant cytotoxic drug like bleomycin results in a very precise and selective destruction of the tumor cells. Thus, in the preservation of the normal cells and tissues structures, dramatically reducing the side effects of the classical chemotherapy and of the ablative procedures. As such, ECT is an efficient, easy to apply, and safe new method for precision medicine. In particular, ECT precision allows to efficiently and securely treat the margins and surroundings of the tumor mass because the few tumor cells present in this volume will be eliminated while the normal cells in the same volume will remain viable. This precision in killing replicating cells, i.e., tumoral cells, explains why ECT spares healthy tissues and preserves organ function, which in certain patients is the most important aspect for choosing a treatment, particularly for preserving life quality. This selectivity confers the treatment reduced side effects, which are mild and self-limited, and even provides very good cosmetic results in the treatment of cutaneous and subcutaneous tumors (28). It is also noteworthy that, even in the case of tumors that have genetic characteristics that confer resistance to multiple drugs, and even to radiotherapy, ECT still plays an important role due to its capability to overcome these barriers. ECT forces the drug into the cells, achieving excellent results where other treatments had failed. Finally, not only the electric pulses per se are an immunological adjuvant as demonstrated in (23), but moreover, ECT causes immunogenic cell death (29), an important aspect of the success in cancer precision medicine.

Many times in the veterinary setting, ECT is performed following standard procedures made for human patients, and that reduces its effectiveness. ECT in vet medicine should be performed considering the unique characteristics of the patients and taking into account the differences among the species that are treated. To achieve its maximum potential adequate treatment planning is crucial. The proper selection of the electrode will depend on the anatomy of the patient, tumor location, invasion depth, and size of it. Also, it is very important choosing the right drug, and the adequate way of administration.

In this work, we report the Veterinary Guidelines for Electrochemotherapy of superficial tumors. Based on published data in peer-reviewed journals and enriched with a careful compilation of the users' questions, and common difficulties. We report a list of potential deviations that can reduce treatment efficacy, and we provide ways to attenuate their consequences. The prevention of these deviations in the practice of the ECT is as important as the accurate application of these guidelines.

## Materials, Equipment, and Patients

### Patients

The main goal is to provide cancer patients with the best treatment available and maximize their chances of success.

In selected patients, ECT alone can be a very good treatment option which can provide excellent results. However, in veterinary medicine, it is common to receive patients in very advanced stages of the disease, with large tumors where ECT alone may not be effective or will require too many sessions. In these cases, combining ECT with other treatment modalities is very effective as will be described later. ECT can thus be used alone or as a neoadjuvant, adjuvant, or concomitant treatment with surgery, chemotherapy, or radiotherapy (25, 30). Adequate treatment selection, based on the patient's oncological situation, is crucial for obtaining the best results.

The owner should understand the expectations of the selected treatment, and the alternatives, and after that, sign an informed consent.

### Indications of ECT

1. Cutaneous or subcutaneous tumors (primary or metastatic) of any histology, which cannot be satisfactorily treated with their respective first-line treatments (26, 31).
2. Primary or metastatic tumors affecting the quality of life due to bleeding, ulceration, or pain (26).
3. Oral or Nasal tumors, as a single treatment, or in combination with surgery (8, 9, 32).
4. Incompletely resected tumors (including surgical scars, skin flaps, and other surgical repairs), or for extending safety margins during surgery (33).
5. Elective ECT treatment when other first-line therapies are possible. A thorough explanation of other treatment options had to be made, and they had to be rejected by the owner (31, 34).
6. Reducing cancer burden in primary or metastatic tumors that are accessible to the electrodes (superficial or by surgical approach) (26, 35).

- In patients under systemic therapy, for the treatment of the lesions that do not show good response.
- In large tumors before surgery, to improve relapse-free survival.
- In patients without treatment options, to improve quality of life in a palliative intent.

### Pre-treatment Tests and Considerations

Mandatory pre-treatment tests are listed below.

1. Pregnant or lactating animals; special care should be taken with unspayed females as bleomycin and cisplatin can harm the developing fetus.
2. Blood tests to determine kidney and liver function should be within normal parameters. Hematological and coagulation parameters should be normal and comparable to those required for a simple surgical procedure.
3. Anesthetic risk including cardiological examination should be evaluated.
4. Histopathological diagnosis of the tumor.
5. Complete oncological staging including three-view chest radiographs, abdominal ultrasonography, CT scan, or MRI if needed, to evaluate the presence of metastasis or other comorbidities.
6. According to each specific case, pain management may be required before ECT and may be adjusted thereafter.
7. Assess the risk in patients who previously received bleomycin; the maximum cumulative dose for dogs is 200,000 IU/m^2^, while for cats it is not yet established (36). Patients who reached this dose should be treated with cisplatin (except cats) or calcium administered intratumorally. If this is not possible, consider another treatment option.
8. History of allergy or hypersensitivity to bleomycin or cisplatin.

In dogs and cats, special attention should be paid to the following cases, as the treatment can be difficult to perform, and the chances of success are reduced.

1. Tumors in, or close to the larynx. Before initiating the anesthetic procedure, make sure that the lesion does not impede the intubation. Consider that after the treatment, the lesion will swell, and may obstruct the airway. If the lesion is large and blocks a considerable part of the airway, debulk it and treat the tumoral bed. Temporary or permanent tracheostomy may be an option for reducing the risk of airway obstruction after the treatment and during the recovery.
2. Tumors in the tongue. The tongue has terminal irrigation, and a very broad application of the electric pulses might induce necrosis of its rostral part, due to the vascular-lock phenomenon. If the tumor is located caudally, it may obstruct the airway after the treatment. In both cases, consider surgical debulking followed by ECT in the tumoral bed. A feeding tube may be needed in the early recovery days.
3. Tumors that compromise the cribriform plate, the retro-orbital region, paranasal sinuses, or other unreachable structures. Tumors located in the caudal region of the nasal duct may invade the ethmoidal cells, which cannot be treated successfully using standard electrodes as they are very difficult to reach. This will ultimately lead to treatment failure. Consider other treatment modalities like surgery plus radiotherapy.

Special considerations for treating horses.

1. They must be treated under general anesthesia, and they must have the appropriate pre-surgical examinations for the procedure. Tetanus vaccine or hyperimmune serum can be administered at the discretion of the veterinarian.
2. The use of the IV route is costly considering the high volumes of drug required, and there are not enough safety studies in this species. Also, the use of intravenous cytostatics should be avoided in animals that may be destined for human consumption.
3. The most frequently treated neoplasias are sarcoids, cutaneous malignant melanoma, and squamous cell carcinoma, all of them with good results (19, 37). Other histologies can be treated whenever their approach is feasible using the available electrodes.
4. The treatment of ulcerated lesions must be adapted to the living conditions of the animal and may require the administration of antibiotics and/or repellants.

### Assessing Size and Number of Tumors

To determine the best treatment strategy, count and measure all the lesions. It is recommended to take pictures including a ruler, to document response to the treatment. They should be taken at the same angle and perspective every time. Also, other imaging procedures may be used to document the effect of the treatment.

For calculating the tumoral volume use the following formula,


$$\begin{array}{l} Tumoral\;volume\;\left[cm^{3}\right]=a\;\cdot b\cdot c\cdot \frac{\pi }{6} \end{array}$$

Where a, b, and c are the length, width, and thickness of the tumor, respectively. For thin tumors (or when lesion thickness cannot be measured) replace the c by the b, as can be seen in the following formula,


$$\begin{array}{l} Tumoral\;volume\;\left[cm^{3}\right]=a\;\cdot b^{2}\cdot \frac{\pi }{6} \end{array}$$

The tumoral burden should be put in context to the size of the animal treated, however, the size of the tumor by itself has an impact on the treatment outcome.

At this point, it has to be determined if the ECT will be performed as a palliative or curative intent, and the number of sessions needed for that end.

#### ECT as a Curative Intent

Tumors of up to 3 cm^3^ can be treated easily with good results, whatever their shape. It can be used as an alternative to surgery when it is difficult, to avoid postoperative complications, or when the tumor is close to important structures (38–40).

In the case of extensive superficial tumors, of <1 cm thickness, even if they are larger than 3 cm^3^, ECT is a good option when first-line treatment modalities have failed or if they are not feasible (38, 41–43).

**Figure 1:**
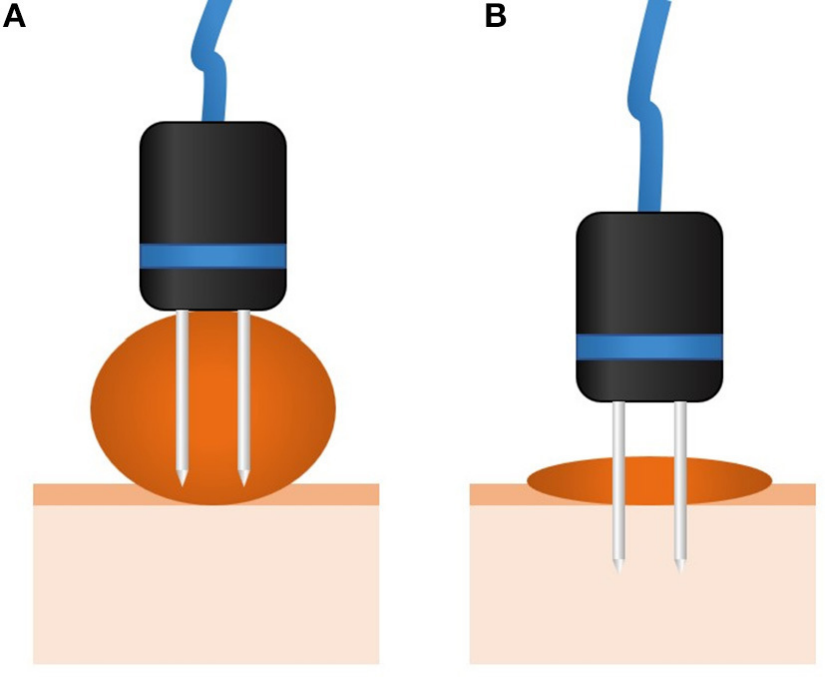
Scheme showing the treatment of a tumor thicker than the needle's length. In **(A)**, as can be seen, in a thick tumor the tumoral bed is not treated, leading to a relapse. In these cases, surgical debulking is recommended to achieve the situation in **(B)**. Otherwise, the same situation in **(B)** can be achieved by multiple ECT sessions, which should be already planned when the patient is evaluated for treatment.

Special care should be taken with the thickness of the tumor, as the length of the needle of the electrode could limit the possibility of treating the tumoral bed. Please, note that only the conductive part of the needles should be considered (some devices use electrodes that despite the needle being long, some part of it is isolated). Tumor thickness should be less than the length of the needle's conductive part, which usually varies from 1 to 4 cm, depending on the manufacturer. If the tumor is thicker than the conductive needle's length, debulking followed by ECT of the tumoral bed and margins is recommended. If debulking is not performed, a second session of ECT must be scheduled to complete the treatment, and performed after the response to the initial session is achieved. These additional rounds of ECT are part of the treatment planning, and should be established during the patient's initial evaluation (44). See Figure 1.

#### ECT as a Palliative Intent

ECT is a very useful treatment strategy, and in this context, superficially extensive or large tumors can be treated to improve the quality of life, provided there are no curative alternatives (45).

### Owner Information and Informed Consent

**Figure 2:**
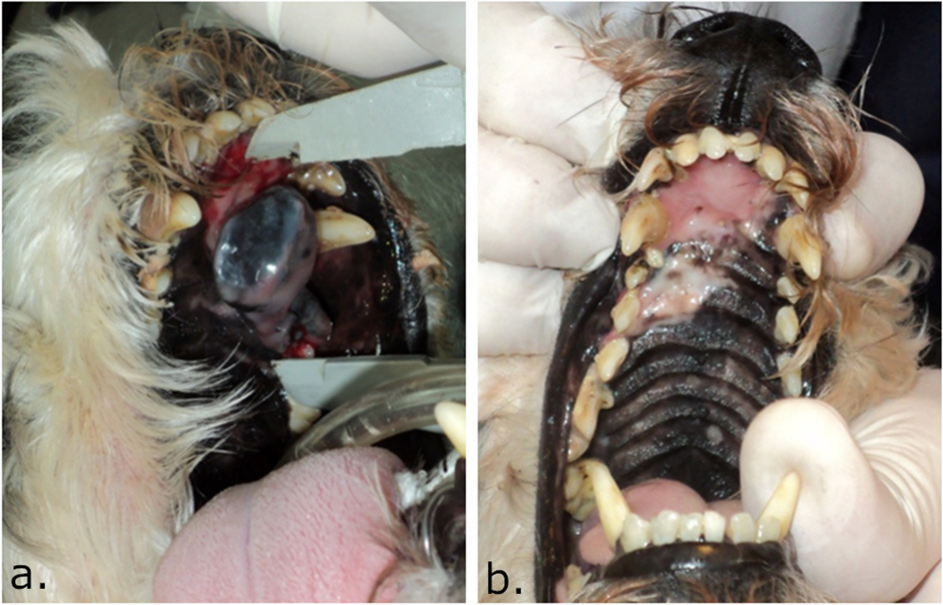
Picture of the palate of a dog treated with ECT using IV bleomycin where induced hypopigmentation can be seen. In **(a)**, the dog with a melanoma in the palate before the treatment. In **(b)**, 4 months after ECT, a complete response was obtained and a hypopigmented area is seen where the tumor was located. Note that as the bone was not compromised, the palate integrity was preserved. In some cases, hypopigmentation may partially revert with time.

Owners should be informed about all treatment options, the benefits and drawbacks of ECT, and its possible side effects. Owners should also be informed about the expected outcome of the treatment. Once these issues are understood, a proper informed consent should be signed by the owner, which should include: (i) the risks associated with the drug used for the procedure, i.e., lung fibrosis when using bleomycin in a patient with a high accumulated dose, and risk of alopecia and/or changes in the pigmentation of the treated skin (see Figure 2), (ii) the risk of pain after the procedure, for which an adequate management plan has to be provided, (iii) the risk of formation of a fistula, extensive necrosis, or serious post-treatment anatomical defects when the tumor compromises the whole thickness of the tissue, that may require reconstructive surgery, as well as (iv) the possibility of needing more treatment sessions in certain cases.

### Electroporators and Pulse Parameters

The standard accepted pulse parameters for ECT are 8 monopolar square-wave pulses of 100 μs, delivered at a repetition frequency of 1–5,000 Hz, with a voltage-to-distance ratio of 1,000–1,300 V/cm, depending on the electrodes used. The repetition frequency does not affect the response. When frequencies higher than 100 Hz are used, only one muscle contraction is seen improving tolerance, and comfort for the patient (46, 47).

Monopolar square-wave pulses are the preferred type of pulses for ECT. However, many configurations of bipolar pulses have also displayed good results (34, 48). Further research is needed to determine if there are benefits of using bipolar pulses in gene electrotransfer or ECT (24, 49).

Many electroporators are available for performing ECT in veterinary medicine. Some of them are for laboratory use, and others are specifically designed for their use in veterinary clinics.

Automatic devices are preferred since configuration errors are prevented. The electroporator should be able to maintain the voltage (without drops) in each one of the 8 pulses of the train. That is possible if the generator can deliver an adequate maximum output current. According to our experience, current outputs of 35 A (during the 100 μs of the pulse duration) are enough for treating all kinds of tumors.

It is very important to always use the electrodes provided by the manufacturer of the device. The basis for choosing the best electrode for a patient is discussed in the following section.

If a configurable electroporator is used, it should be properly set. To this end, verify the distance between the needles of the electrode that will be used and multiply this distance in cm by 1,000 to obtain the voltage to be used. For instance, if you are using needle electrodes separated 0.4 cm from each other, you have to set the device output voltage at 400 V (1,000^*^0.4 = 400). In the case of plates applied on the skin, you should multiply the distance by 1,300. Thus, in the same example, you have to set the device output voltage to 520 V (1,300^*^0.4 = 520). In the case of plates applied directly onto the tumor, use 1,000 V/cm, like for the needles. Then set the pulse length to 100 μs, and the pulse interval between 100 μs and 1 s (for a repetition frequency between 5,000 and 1 Hz). Set the device to deliver 8 pulses (50).

### Drugs Used

For performing ECT only 3 drugs have been validated; bleomycin administered intravenously or intratumorally, cisplatin intratumorally, or calcium intratumorally. The decision basis for the use of each of them is presented in the following section.

## Methods: Treatment

### Anesthesia

The treatment should be performed under general anesthesia, even when using plate electrodes. The sensations and muscle contractions induced by the pulse delivery can be painful and stress the patient, eliciting a pain-induced aggression (51).

The anesthetic procedure required for ECT is similar to the one required by surgery in the same region. It should be chosen according to the expertise and familiarity of the professional who is going to perform it.

As an example, for dogs and cats, a typical regime would be as follows: (i) premedication with intramuscular administration of xylazine 0.5 mg/kg and tramadol 2 mg/kg; (ii) induction made with intravenous administration of propofol 3 mg/kg; (iii) maintenance performed with inhaled isoflurane 2–3 % and intravenous fentanyl 2 μg/kg. Regional anesthesia may be added, according to the criterion of the anesthesiologist, to improve patients' comfort and reduce the dose of the anesthetic drugs. For instance, infraorbitary nerve block can be performed when treating the nose. Anti-inflammatory drugs can be used during or after the procedure and maintained the following days as it will be seen later.

In horses, general anesthesia is always recommended. A typical regime would be combined regional-general anesthesia, to reduce the depth of the anesthetic plane required (52). The induction should be carried out in a safe and carefully chosen environment. The patient should be under the proper analgesic plan during the whole procedure.

In small animals, post-anesthetic observation until the patient is fully awake is very important, especially when the nose or the mouth is treated.

### Drug Administration

Begin by weighing the patient. Then, measure the lesions to calculate the tumoral volumes. After the measurements, proceed to trim the fur of the area to be treated to have a good visualization of the tumor and its margins. Use an iodine solution for cleaning and disinfecting the whole area to be treated (tumor and margins). It is highly recommended to use sterile electrodes or to sterilize them following recommendations from the manufacturer, even though the risk of infection after ECT is very low (53).

For the dilution and administration of the antineoplastic drugs, it is mandatory to wear gloves and a laboratory coat, to use face and respiratory protection. If possible, it is recommended to work in a class II laminar air flow cabinet (54).

#### Intravenous Route

We recommend using intravenous bleomycin for all cases, regardless of the size of the lesion (only bleomycin can be used through the intravenous route, as it is explained later). This recommendation is based on several reasons: (i) an adequate distribution and concentration of the drug are almost always achieved in the tumor and its margins when the timing of administration is observed, (ii) avoids leaving areas of the tumor with insufficient drug concentration due to errors in the intratumoral administration technique, (iii) drug administration is safer, as spilling and leaking that can occur during intratumoral administration are avoided, and (iv) by itself is an immune system activator, contributing to the local immune response induced by the treatment (23).

Note that intravenous bleomycin administration is not recommended for horses due to the huge volume of drug needed.

The potency of bleomycin is measured in units of antimicrobial activity. In many countries, bleomycin is either dosed in mg or International Units (IU), whereas Units (USP) is the term used in the USA. The equivalence would be 1 USP unit = 1 mg (by potency) = 1,000 International Units (IU) (55).

Use the weight of the patient to estimate the body surface area (BSA) with the following formula.


$$\begin{array}{l} Body\;Surface\;Area\;\left[m^{2}\right]=k\cdot {weight\left[kg\right]}^{2/3} \end{array}$$

Where *k* is 0.101 for dogs and 0.1 for cats (56).

##### The Technique of Administration for the Intravenous Route

The drug is administered at a dose of 15,000 IU/m^2^ BSA in bolus (in 30–45 s), and the maximum dose is capped at 30,000 IU (corresponding to 2 m^2^ BSA) (50). The application of the electric pulses can begin 5–8 min later, after the drug has diffused into the tumoral tissue.

After administration, approximately half of the Bleomycin dose administered is eliminated by renal excretion, so a reduced dose can be used in patients with decreased renal function (57, 58). Remember that the maximum cumulative dose for dogs is 200,000 IU/m^2^, while for cats it is not yet established (36), to avoid bleomycin's induced lung fibrosis, its major side effect. The drug is metabolized in tissues by the bleomycin hydrolase enzyme, which is in very low concentration in the skin and lungs, explaining the sensitivity of these tissues to bleomycin toxicity (58).

The great difference in sizes and body weights among veterinary patients needs to be considered when estimating the treatment window. This time can be affected by many factors, among them are the tissue blood flow rates (59) which are influenced by the heart rate. As is known, heart rate is related to body weight (60), so we can arbitrarily consider a treatment window closer to 5–25 min for cats and small dogs, and 8–40 min for the rest. Similarly in human medicine, patients younger than 65 years old have a treatment window of 5–15 min, vs. an 8–40 min treatment window for older patients, this difference is related to the kidney function (61). Further study of bleomycin pharmacokinetics is needed to properly define the treatment window in patients of different species and body weights.

The optimal efficacy for applying the pulses is obtained up to 40 min after the administration of the drug. However, this time can be extended in elder patients as well as in patients with impaired renal function. In any case, it is recommended to continue the application of electric pulses to the remaining lesions even after that time, as there is still an effect on the lesions. It is advisable to mark these lesions, to recognize them during the follow-up (57, 62).

#### Intratumoral Route

The intratumoral administration is acceptable for small tumors of up to 2 cm^3^ (63). In large tumors, this route can be challenging, in particular in horses and cats. On the contrary, the intravenous route will provide an adequate distribution of the drug in all cases, and for that reason it should be preferred whenever possible.

Bleomycin, cisplatin, and calcium can be administered intratumorally. The delivery of the electric pulses should begin immediately after its administration.

##### Bleomycin

The recommended concentration of bleomycin for intratumoral administration is 1,000 IU/ml, and the dose is 250 IU/cm^3^ of tumor (50).

The tumor must be completely infiltrated with a total dose lower than the one that would be used in the intravenous route. If this is not possible, use the intravenous route of administration under the conditions described in the previous section.

In unusual species, like turtles, snakes, or birds, among others, the intratumoral route is preferred due to the lack of information on the dosing and effects of systemic bleomycin in these animals.

##### Cisplatin

When using cisplatin, the recommended concentration is 1 mg/ml, and the recommended injection dose is to fill the tumor volume with the drug (50). In case cisplatin is not available at the recommended or superior concentration, we suggest using bleomycin. The use of a lower concentration is possible but requires a close follow-up, as the patient may need retreatment (64).

Cisplatin is a good option for treating horses (11, 19). On the contrary, this drug is not recommended in cats (65).

Carboplatin can be used if cisplatin is not available, however, its effectiveness has only been demonstrated *in vitro* (66).

##### Calcium

When using calcium chloride, the recommended concentration is 9 mg/ml. Inject a volume of calcium chloride solution equal to half of the volume of the tumor (67, 68). Electroporation with calcium can provide good results and can be used when bleomycin or cisplatin are not available, as the third option. It should also be restricted to small tumors (67).

##### The Technique of Administration for the Intratumoral Route

Insert the needle at a single point in the center of the tumor, and radially administer the drug to avoid spilling. When injecting the drug, pay attention not to remove the needle too quickly to avoid spilling or leaking the medication. As only small tumors should be treated with this technique, safety margins are covered by drug diffusion from the lesion providing an adequate concentration for successful treatment of the margins. Healthy tissue, if infiltrated by any of the previous drugs can necrotize, and thus direct injection of the surrounding healthy tissue should be avoided (69).

The electric pulses should be applied immediately after the administration of the drug, as it washes out quickly (50). If there is more than one lesion to treat, it is recommended to administer the drug and deliver the pulses to them, one by one. It is important to point out that if the drug is administered to all tumors in the first place, and the pulse delivery is performed after that, the last ones to be pulsed may not have an adequate drug concentration.

In horses, where intravenous bleomycin is not possible, large tumors can be treated in this way; inject half of the lesion and pulse it immediately after. Then, inject the other half and pulse it. By doing this, a large lesion can be successfully treated.

### Electrode Selection

#### Electrode Types and Their Advantages

**Figure 3:**
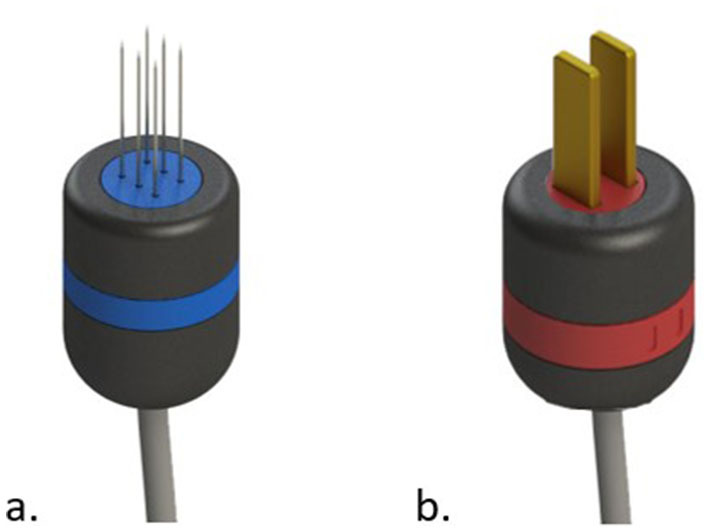
Common types of electrodes for electroporation. In **(a)** needles electrode. In **(b)** plate electrodes.

In general most devices come with two types of electrodes; the needles electrode, and the plate electrodes (see Figure 3).

Always use electrodes provided by the manufacturer of the device and observe the maximum number of uses intended for them. Exceeding the maximum number of intended uses may significantly reduce the effectiveness of the treatment. If available, disposable electrodes are recommended.

##### Needles Electrodes

This type of electrode is recommended for the treatment of the great majority of tumors.

In veterinary patients, the skin is thicker than in humans (70), being a very resistive layer that can interfere with the homogeneity and the intensity of the electric field at the tumor level. Moreover, skin thickness and thus skin electrical impedance is very variable, depending on many factors, i.e., the species, the race, the age, and the part of the body, among others. By using needles, this thick layer is surpassed, and the electric field can be applied with an adequate distribution. Therefore, needle electrodes are always preferred.

The needles are usually distributed in two rows of three or four, separated 0.4–0.5 cm from each other. There are other patterns, like the hexagonal, that behave in the same way.

**Figure 4:**
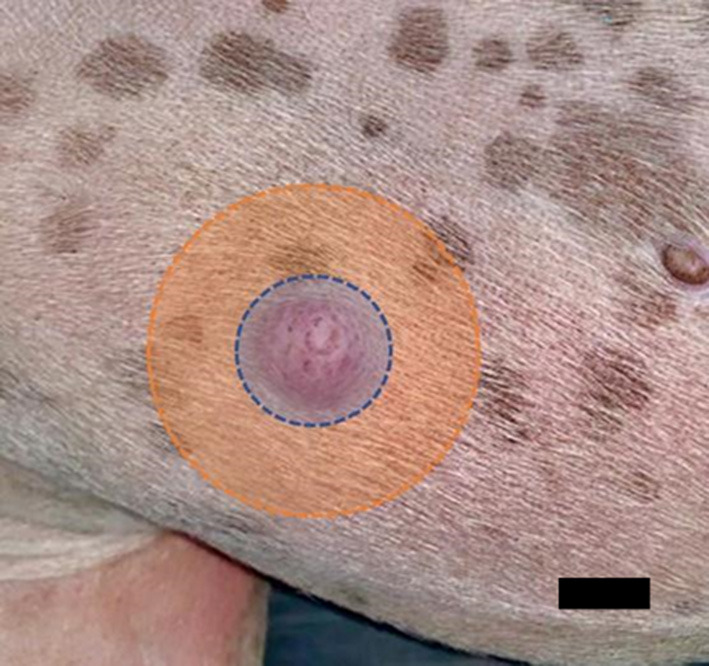
Principle of treatment for mast cell tumors. First, the periphery should be treated (orange area), and then the tumor (blue area). Scale-bar 1 cm.

The whole tumor surface should be covered to adequately treat the tumor. In particular, mast cell tumors should be treated spirally from the periphery to the center (see Figure 4). As the electric field drops off very quickly outside of the electrodes, a minimum superposition in the application is needed to avoid leaving untreated areas. If the tumor is thicker than the length of the needles, surgical debulking may be needed. Also, it can be treated in multiple sessions, which should be at least 4 weeks apart, to avoid overtreatment of the area, provided there is no growth of the tumor. If the growth of tumoral tissues is seen, the following treatment session should be performed as soon as possible. A particular case is when the tumor is only partially treated in the first session. In this case, the remaining untreated tissues can be treated the following day without concern.

For the decision basis on when to retreat a previously treated tumor, please see section Follow-up and Retreatment.

##### Parallel Plates Electrodes

**Figure 5:**
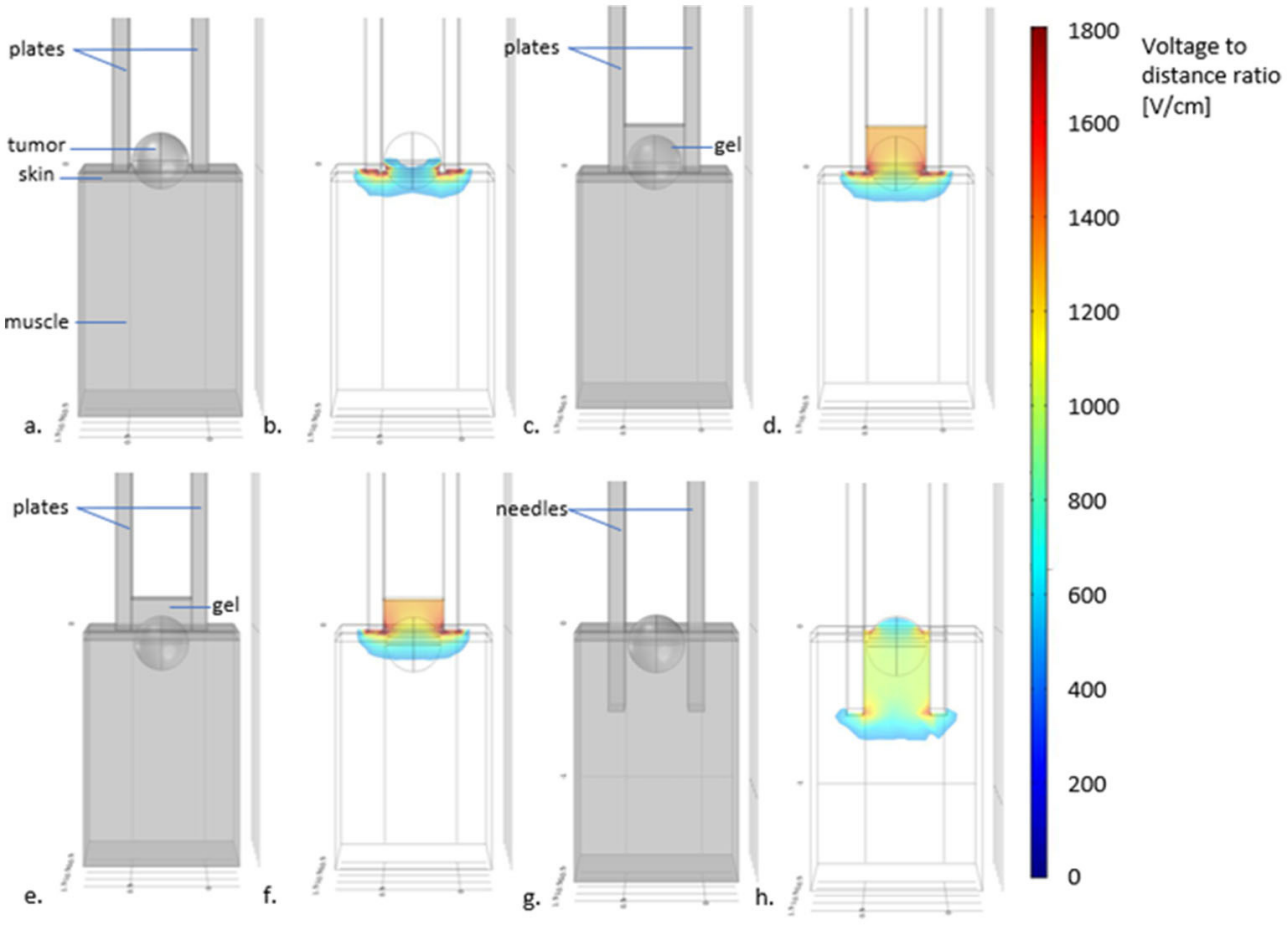
Scheme displaying the depth of treatment with different electrodes and visual recommendations for the use of plate or needles electrodes. In **(a)** plates electrodes used for treating a superficial tumor, without using conductive gel. In **(b)** the simulation of the electric field distribution (using COMSOL Multiphysics 4.3—in color the electric field intensities above the threshold for tissue reversible electroporation) reveals that the superficial parts of the tumor may not be adequately treated. In **(c)** the scheme shows the addition of gel between the plates. In **(d)**, the simulation shows that now, the distribution of the electric field allows treating the tumor completely. In **(e)**, the case of a tumor with an invasion depth >3 mm treated with non-penetrating plate electrodes. As it can be seen in **(f)**, even with the use of gel, the deepest parts of the tumor may not be adequately treated. Indeed, the field intensity drops below the electroporation threshold at a distance lesser than the separation of the plates. For these cases, needle electrodes should be used, as depicted in **(g)** where the same tumor (with an invasion depth >3 mm) is treated with needle electrodes without conductive gel. The electric field simulation in **(h)** shows that the whole tumor is now completely treated.

Parallel plates electrodes are very useful to treat superficial lesions with a few millimeters invasion. The depth of treatment of these kinds of electrodes varies according to the distance between the plates (71). According to the simulations presented in Figure 5, in electrodes with plates separated 4 mm from each other the maximum treatment depth is <3 millimeters. Lesions that invade deeper than this, should be treated with needle electrodes. It is important to remember, that for treating superficial lesions through the skin the voltage-to-distance ratio should be 1,300 V/cm (72). But, for the treatment of the tumoral bed after debulking surgery, 1,000 V/cm is enough. As the skin is removed, the electric field can reach deeper parts of the tissues (71).

Plate electrodes are very useful for treating the eyelids, and ears by putting them in-between the plates.

Other designs work similarly to plate electrodes, such as contact electrodes and L-shaped electrodes. These kinds of electrodes are used for the treatment of superficial tumors only, and tissues cannot be treated by placing them in between the conductive parts, as is possible with plate electrodes. Particularly in some devices, an L-shaped electrode is configured to deliver 4 pulses, and for that reason, orthogonal rotation between two pulse trains is required.

Always monitor the contact between the tissue and the electrodes (73). If it is not adequate, conductive gel should be used to improve the electric field distribution and thus the result of the treatment. The gel should have a conductivity similar to the tissue treated. For general purposes ultrasonography gel is adequate. Remember that an excessive amount of gel is better than an insufficient one to improve contact between the tumor and the electrode (74). Avoid using petroleum jelly as it can impede the flow of the electrical current reducing treatment efficacy. Also, avoid excessive overlapping of two adjacent applications, particularly in healthy tissue, as it may provoke undesired tissue damage or excessive necrosis (see also section Actionable Recommendations).

#### Safety Recommendations

The electroporator used should comply with basic safety standards, including arc detection, short-circuit detection, and voltage drop alarm to ensure an adequate pulse delivery.

**Figure 6:**
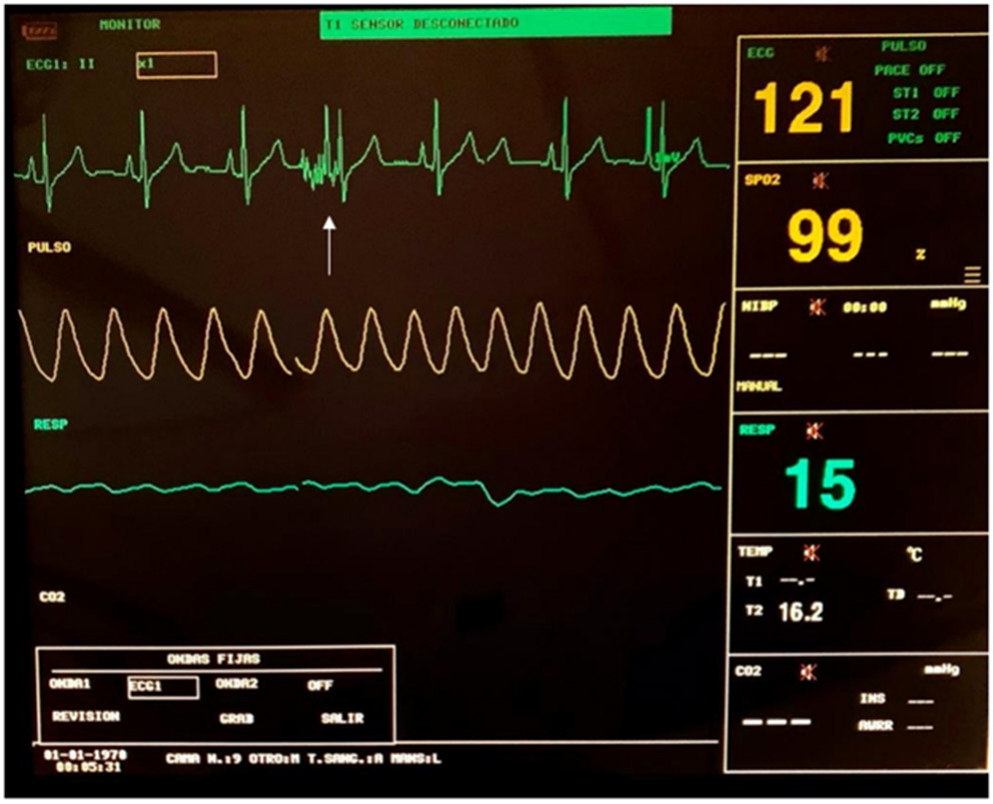
ECG monitoring during an ECT procedure. An artifact can be seen during the delivery of the pulses (white arrow).

Artifacts in ECG monitors may be seen during pulse delivery, due to electrical interference between devices, and they should not be mistaken for arrhythmias or any other cardiac alteration (see Figure 6).

Metallic surgical instruments should be kept away from the electrodes, and the area of treatment, avoiding their contact during pulse delivery. This point will be addressed in section Actionable Recommendations.

### Antibiotics and Analgesia After the Treatment

Prophylactic antibiotics can be administered orally, or intramuscularly, before or after the procedure.

The use of non-steroidal anti-inflammatory drugs (NSAIDs) is the recommended option for pain control, because, after the treatment, pain can be provoked by the inflammation of the treated tissue. In large lesions, the association of NSAIDs with opioids may be useful. If the treatment is on the nose, tongue, eyelids, or close to the larynx, the use of corticosteroids is preferred over NSAIDs during the first 48 h, due to their greater anti-inflammatory effect. NSAIDs can be used after the suspension of the corticosteroids. However, simultaneous use of both, which is common in human medicine, is counter-indicated in dogs and cats as it increases side effects (75).

### Wound Care

No dressing is needed for the wound after the treatment. In the subsequent days after the treatment, the treated area may present oozing that can be cleaned by the owner. Elizabethan collars can be used in cats and dogs to prevent the animal from licking the treated area.

## Evaluating the Results

### Follow-Up and Retreatment

Follow-up is planned individually depending on patients' needs and is recommended at 15 days, and at 1, 2, 4, and 6 months after the treatment. At each follow-up, the lesion should be measured and photographed to document the response to the treatment.

Once the tumor is treated, it slowly reduces in size with little or no necrosis. The mechanism of action of bleomycin consists of cutting the DNA strands, and the cells die when trying to divide. For this reason, tumoral cells are “marked” to die, but only die after they try to divide (58, 62). As long as the lesion keeps shrinking, no more treatment sessions are needed, because there is no benefit in treating already treated cells. On the contrary, it may induce necrosis of the tissue. The maximum therapeutic effect is seen after 6–8 weeks, but it may take longer. Sometimes, the tumor enters a quiescent state, and after 2 or 3 months starts shrinking again.

Before scheduling a new treatment session, the full response should be awaited, instead of performing the next session on a fixed time basis. This applies also to the retreatment of a previously treated lesion. Note that in the case of previously untreated tissues or parts of the tumor that were left untreated (in the case of very large lesions, for example in horses) there is no need to delay a new session to treat these untreated tissues. In the case that the treated lesions prove to grow again, the new treatment session should not be delayed.

Even though tumor lysis syndrome is very uncommon, when treating large tumors special measures should be taken to prevent it, in any case, a quick diagnosis and prompt treatment is essential.

**Figure 7:**
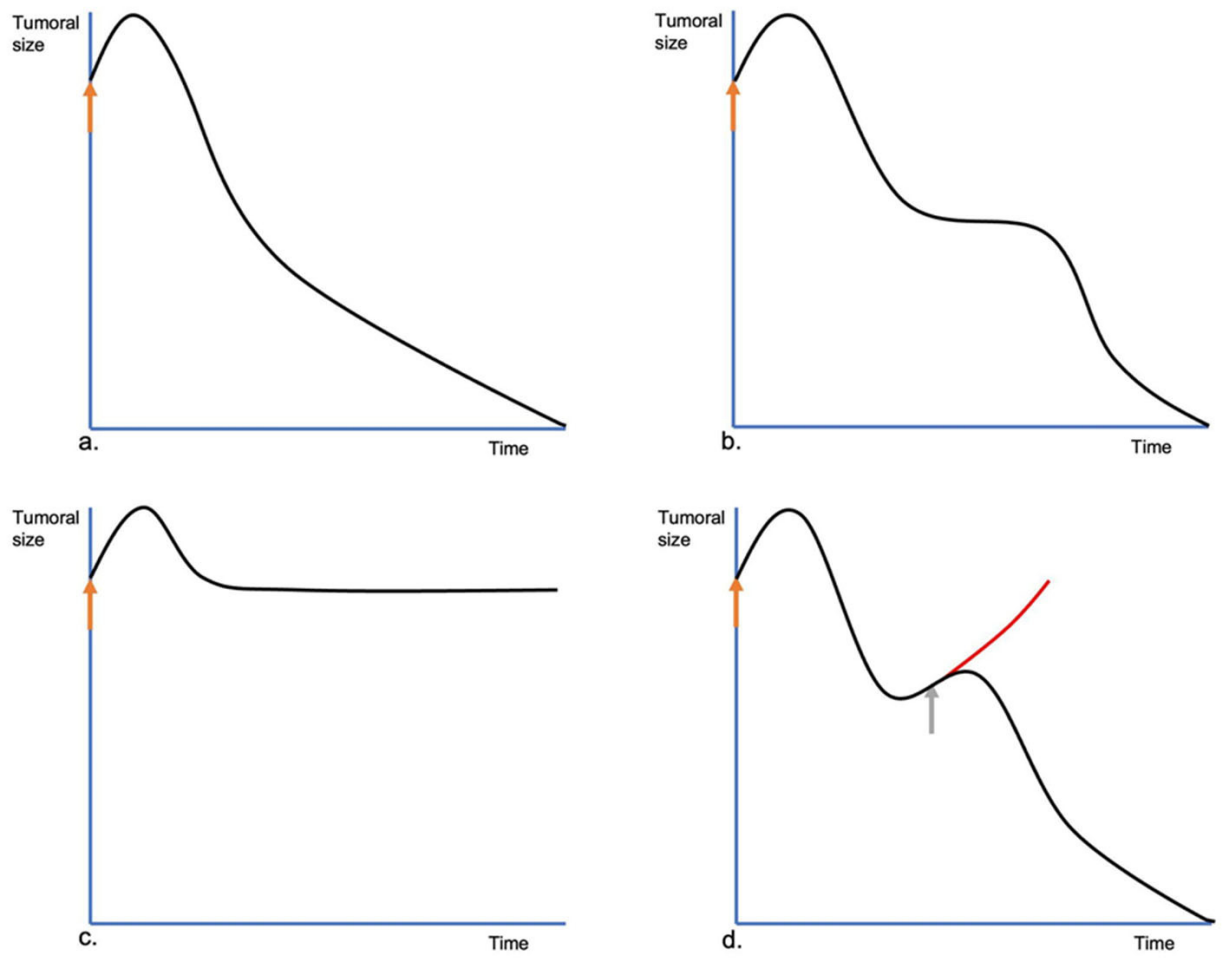
Different evolutions of the tumors treated by ECT. In **(a)**, the common evolution. At first, immediately after the ECT session (orange arrow), the treated lesion swells, increasing its size, but on the following day, it starts to reduce steadily until its complete remission. In **(b)**, the two-times evolution. The lesion, after the initial swelling, starts shrinking, but it stops. The lesion remains steady for some time and then resumes shrinking until its final response. In **(c)**, the no-change evolution. After the initial swelling, the tumor shrinks to its pretreatment size, without shrinking any more, or shrinking very slowly. In **(d)**, the tumoral escape evolution. After the initial response, the tumor starts growing again. A new ECT session should be performed as soon as possible (gray arrow) to prevent a relapse.

**Figure 8:**
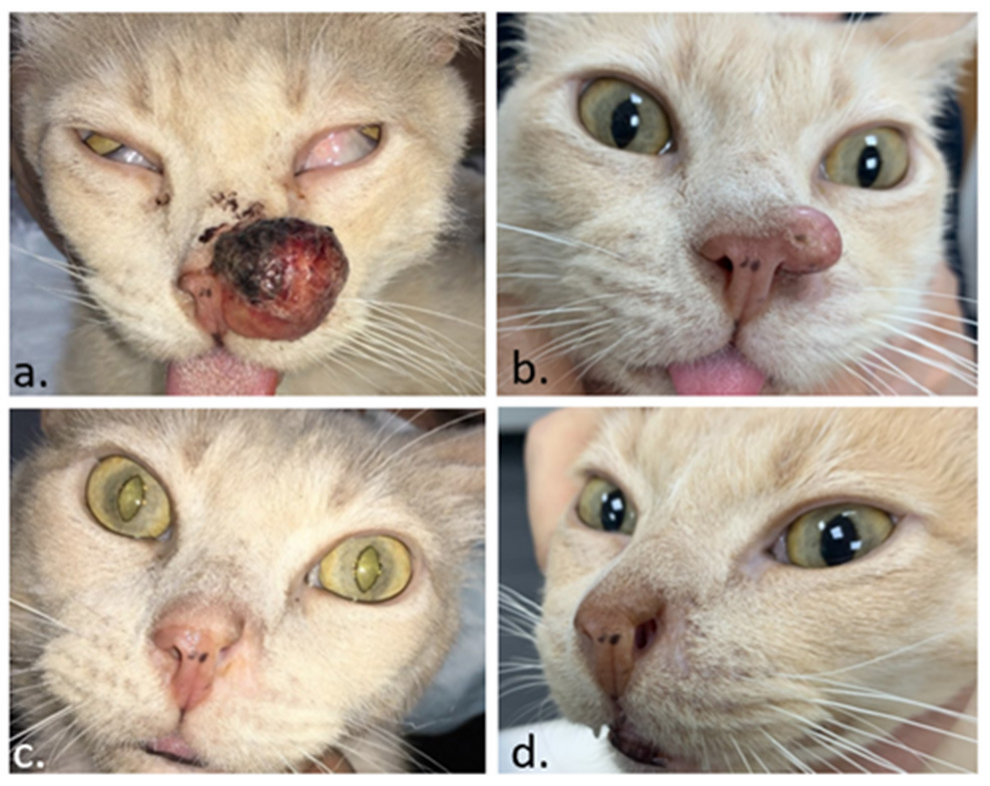
Feline patient with a squamous cell carcinoma in the nose. In **(a)** the day of the ECT. In **(b)** 1 month later, the tumor regrew, and a second ECT session was performed. In **(c)**, a complete response was obtained 1 month after the second ECT. In **(d)**, the patient remains disease-free 4 months later.

In the follow-up of a tumor treated with ECT, four kinds of evolutions can be seen. The *common* evolution is what happens in most cases. The tumor shrinks after an initial swelling and keeps shrinking until the final response is achieved (Figure 7a). Sometimes, in the beginning, the lesion behaves like the previous, but shortly after, it stops shrinking. It stays the same size for a variable time, then it resumes shrinking. We call this a *two-times evolution* (Figure 7b). This could be attributed to the non-dividing tumoral cells, which enter the cell division cycle and die only at that moment since they have their DNA strands cut by bleomycin without killing them immediately. Another type of evolution is characterized by lesions that after the treatment do not show an evident shrinking, due to their high population of quiescent cells (see Figure 7c). We call this *no change evolution*. The *tumoral escape evolution* is seen when after the initial reduction, the tumor starts growing again (see Figure 7d). In this case, a new treatment session should be scheduled with no delay as the tumor has been insufficiently treated (see Figure 8). It is important to note that the tumor evolution is different from the response, as the response can be the same in the four cases. Understanding the types of evolution is essential to determine whether to repeat a treatment session or not. In the first three types of evolution, it is recommended to wait and make a close follow-up of the lesion. Especially in the two-times evolution, or in the no-change evolution, as they may end in the tumoral escape evolution. This is particularly important, as the treatment of lesions that are evolving following one of the first three types of evolution, may be unnecessary and can even lead to tissue necrosis, due to overtreatment. If there are doubts about a steady lesion, its recommended to perform a biopsy to avoid confusing a remaining of the tumor with residual scar tissue.

There is no data regarding minimum intervals between retreatments, but at least 4 weeks is a period recommended when using intravenous bleomycin.

Consider retreatment if

- The lesion was not sufficiently treated in the first session.
- The lesion starts to grow
- New lesions develop.

### Combination With Other Therapies

ECT does not preclude other treatments, on the contrary, it may increase their effectiveness. For that reason, many standard treatments such as surgery (10, 44, 51), chemotherapy (30), immunotherapy (24, 76), and radiotherapy (77) may be more effective in combination with ECT (25).

For tumors larger than 3–4 cm^3^ ECT can be combined with surgery as a neoadjuvant cytoreductive tool to perform a less extensive surgery, allowing function or organ sparing. ECT can also be used as an adjuvant therapy to clean insufficiently resected margins. And finally, it can be used intraoperatively, to clean the tumoral bed of the resection (10, 25, 44, 51).

Chemotherapy or metronomic chemotherapy can be used adjuvant to ECT. However, considering the beneficial role of ECT as an activator of the immune system, response may be impaired by the immunosuppressive effect of systemic chemotherapy (29). It must be carefully assessed whether the risks outweigh the benefits for this combination of neoadjuvant, concomitant, or adjuvant chemotherapy with ECT (25).

Radiotherapy can be used in combination with ECT, particularly for tumors that invade an area beyond the reach of the electrodes. The other way around is also possible, ECT can be used after radiotherapy to treat radioresistant relapses (77, 78).

**Figure 9:**
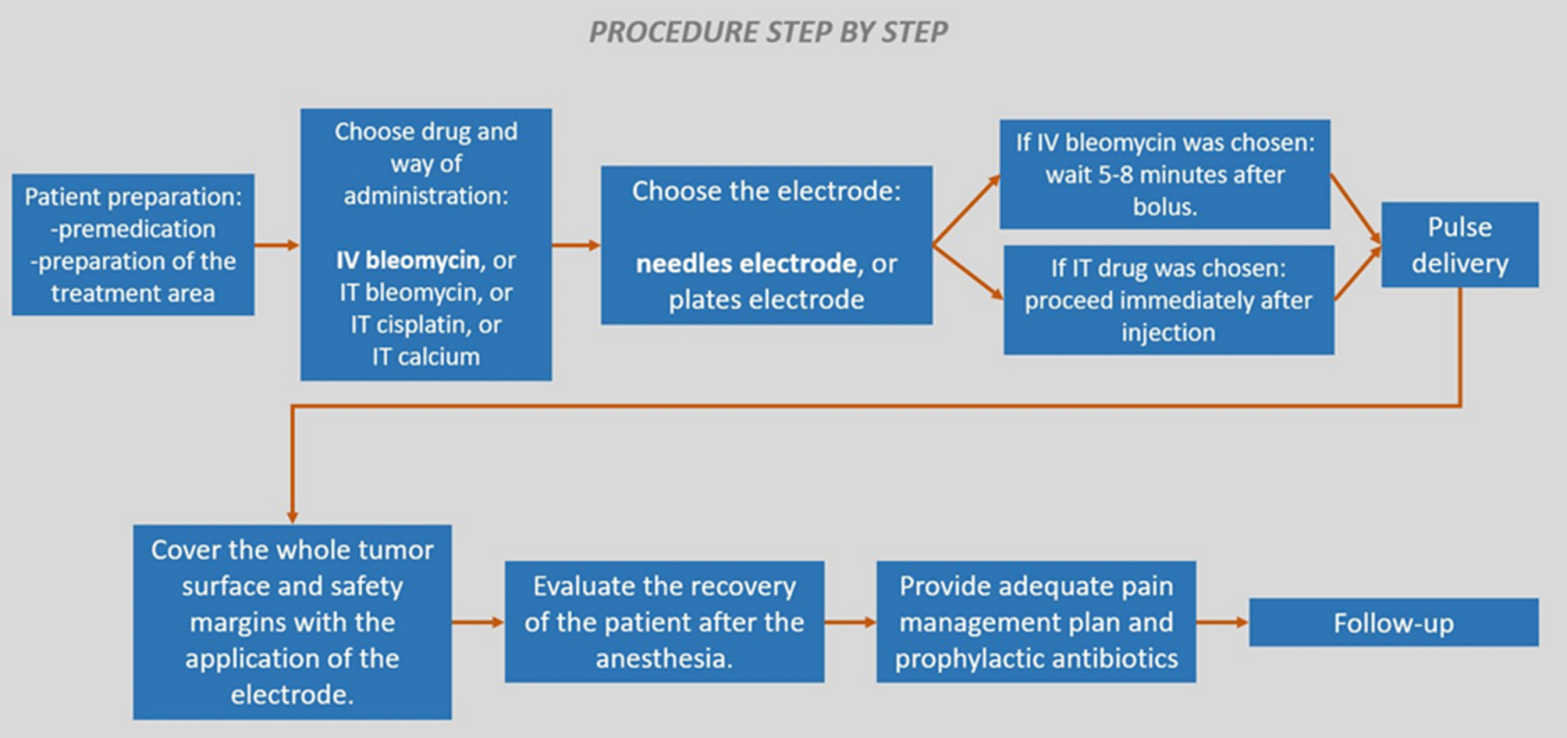
Electrochemotherapy procedure step by step. In bold, the preferred choices when possible.

Finally a procedure step by step on how to perform the treatment is presented in Figure 9.

## Actionable Recommendations: Issues Often Observed in the Procedure That Should be Avoided to Improve Results

### Related to the Patient

#### Issue

Sometimes, too much time goes by between the first consultation and the ECT procedure. In very active tumors, this can completely change the status of the patient, and a re-staging may be needed to confirm that ECT is still the best treatment option.

#### Recommendation

Pre ECT evaluations should be performed a maximum of 4 days before the treatment. If needed, ask for new imaging procedures.

#### Issue

Like the previous one, if a long time goes by between the first consultation and the ECT, even if the tumor did not grow much, the patient can become seriously deteriorated. This is notorious for tumors that impede appropriate feeding. This situation can increase the anesthetic risk, and the procedure might become impossible.

#### Recommendation

Make sure that you will provide appropriate clinical support (sufficient hydration, support in feeding, and adequate analgesia) with the aim that the ECT treatment is performed under the best conditions.

In severely deteriorated patients it is possible to treat only half of the lesion. If its evolution is positive in a few days, then treat the second half of the lesion as soon as possible.

### Related to the Technique

#### Issue

The optimal treatment time is up and remains tissue to be treated.

#### Recommendation

Continue treating all the remaining tissues that should be treated, regardless of the time elapsed. All the lesions treated outside the optimal treatment window should be marked and carefully followed for prompt retreatment in case of regrowth or absence of response. It is advisable to plan the timing of the administration of bleomycin, especially in patients with very extensive lesions, or when the procedure is performed intraoperatively.

When using intratumoral administration of the drug, the injected tumor volume should be treated as quickly as possible. If the tumor is large and the injection lasts more than 2 min (19), deliver the pulses in the injected part and after that, proceed to inject and pulse the other parts.

For more details please refer to section Drug Administration.

#### Issue

The tumor is thick, and the treatment of the deepest parts cannot be assured.

#### Recommendation

The electric field drops very quickly outside the region between the needles or plates of the electrodes. For that reason, special considerations must be made when treating the whole tumor volume including its deeper parts. It is very important to cover the whole tumor, especially its bed, to reduce the risk of relapse. When using surface electrodes, the depth of treatment is around 3 mm maximum. For tumors that invade in greater depth, needle electrodes should always be used to guarantee the adequate reach of the electric field to the deeper tissues (79). If the thickness of the tumor is greater than the needles' length, then proceed with debulking before ECT, as was explained before.

#### Issue

Inappropriate use of the needle electrodes in rounded or pedunculated lesions.

#### Mitigation Procedure

**Figure 10:**
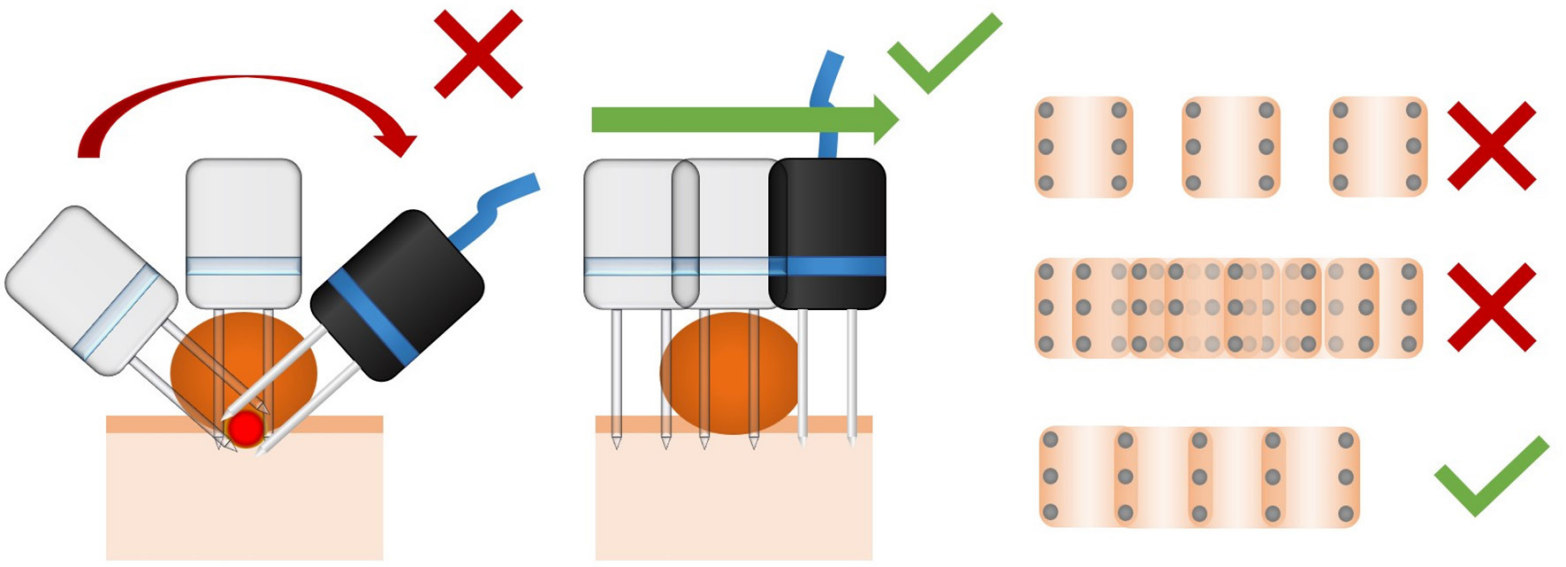
Dos and don'ts about the technique of electric field delivery using needle electrodes. At left, a common mistake during the treatment of rounded lesions is the application perpendicularly to the surface. In this way, the tumoral bed may not be correctly treated, and the center of the mass may be overtreated. In the middle, the delivery of the pulses perpendicularly to the surface where the tumor lies. This provides adequate treatment of the bed of the lesion. At right, is a scheme of the distance between the applications of a 6-needle electrode. As it can be seen, if they are too separated (up), untreated areas may lead to a relapse. If they are too superposed (middle), overtreatment may produce necrosis and unintended tissue damage. The right distance is a slight superposition of the applications (down).

It is very important to ensure the treatment of the whole tumor, carefully avoiding overtreatment. It is of utmost importance to cover the base of the lesion with the electric field. In rounded or pedunculated lesions, a common mistake is to insert the needles electrode perpendicularly to the tumor's surface. This approach overtreats the center of the tumor producing irreversible electroporation, and does not cover the tumoral bed. The correct approach is to insert the needles electrode perpendicularly to the surface where the tumor sits. A slight superposition of the application of the electrode is recommended, and large separation or too many superpositions of the electrode's application should be avoided (see Figure 10).

#### Issue

The tumor is surrounding a tooth.

#### Recommendation

The presence of the tooth between the needles may seriously affect the electric field distribution. The tumor may be treated if it fits between the needles without increasing their separation. But, because the progression of the disease would probably lead to a loosening of the tooth (which will have to be removed later anyway), it is recommended to remove the tooth and treat the area correctly.

#### Issue

Metallic implants or surgical instruments close to the electrodes can conduct electricity through them and provoke a short circuit and damage the device.

#### Mitigation Procedure

Particular attention must be brought, to avoid the insertion of a needle in contact or very close to any metallic implant or surgical instrument.

#### Issue

The presence of blood in the treatment area may increase conductivity and induce an arc or deviate the electric field causing an incomplete/inaccurate treatment of the tumor.

#### Recommendation

Adequate hemostasis is essential during the procedure.

#### Issue

ECT using drugs that are not bleomycin, cisplatin, or calcium.

#### Recommendation

Use only the proven chemotherapeutic drugs for ECT which are; bleomycin, cisplatin, and calcium, according to the prescriptions recommended in section Drug Administration. There are drugs that, in classical chemotherapy, are effective for specific cancers, but the choice of the appropriate drug for ECT does not depend on the histological type. Most of the commonly used chemotherapy drugs have been tested with little or no benefit by adding the electric pulses (80) because these drugs already penetrate the cells without restrictions, even in the absence of any kind of cell permeabilization.

### Related to the Device

#### Issue

The electroporator is not working properly.

#### Recommendation

Always deliver a pulse to the air before anesthetizing the patient to ensure the devices' proper functioning. If the problem persists, try powering it off, and back on, checking the correct connection of the electrode and the pedal to the device, and then repeating the test. If the problem persists, contact the manufacturer.

#### Issue

An electrical power supply breakdown is an unpredicted event that can occur in almost any setting. Most medical-grade devices come with an internal battery that allows them to continue operating. Electroporation devices often do not have batteries and for that reason, they cannot keep working during an electrical power supply breakdown.

#### Recommendation

A regular uninterrupted power supply (UPS) of 750W can provide enough power to complete 4 or 5 treatment sessions depending on your devices' electrical consumption.

#### Issue

Many treatment sessions are required to achieve a response and/or the treatment is difficult due to the loss of sharpness of the needles.

#### Recommendation

**Figure 11:**
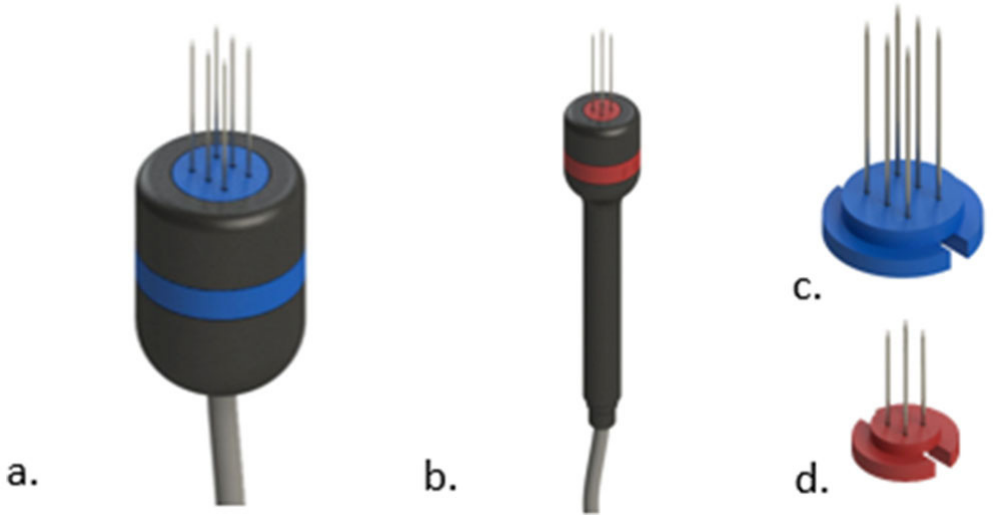
Examples of handles with disposable needles. In **(a)** the handle for the disposable shown in **(c)**. In **(b)** the handle for the disposable shown in **(d)**.

In human medicine, disposable electrodes are the only option for the treatment, but in veterinary medicine, it is still possible to use non-disposable electrodes. Needle electrodes may become seriously affected even after a single ECT session. Oxidation of the needles' surface may isolate parts of them, which can greatly affect the electric fields' distribution and thus reduce treatment efficacy. This oxidation may not be seen in plain sight. Use disposable electrodes if possible (see Figure 11). If they are not available in your setting, maintenance of the electrodes should be made by sanding the needles before the treatment. This will remove the oxidation and improve electrical conduction.

Keep your electrodes in good condition, make regular evaluations and consider acquiring new ones if they show signs of wear.

Needles' sharpness can also be affected, and the ulterior use of these needles may produce unintended trauma to the tissue. Already used needles might also tend to excursion inside the tissue, moving away from each other (which can produce an insufficient electric field) or toward each other (which can produce an arc or a short circuit). If needles are bent, correct the bending before inserting them into the tissue.

#### Issue

The electroporator shows a warning when trying to deliver the pulses.

#### Recommendation

Some tumors display a very high electrical conductivity, which means that very high electric currents are needed to sustain an adequate electric field. The electroporators can be unable to deliver such high currents. If the device that you are using is warning that the electric field was not achieved correctly, remove the needles half the way out and deliver the pulses. Note that the deepest part of the lesion will not be treated, and a new session has to be scheduled as soon as the superficial part (the treated volume) responds. Refrain from modifying treatment parameters as they can induce necrosis and/or seriously diminish treatment efficiency.

## Discussion: The Use of ECT in Veterinary Practice

ECT is a well-established practice in veterinary medicine. It gained status rather quickly because of its high efficiency and negligible side effects, and because it provides adequate treatment when other treatment modalities have failed or are cumbersome.

While ECT is nowadays regularly used in cats, dogs, and equines, patients from a varied spectrum of other species have also been treated successfully, among them: ferrets (81), elephants, fishes, turtles (82, 83), hedgehogs (84), snakes (85), birds (86, 87), pigs (88), among others.

In human medicine, ECT is a very valuable tool. It started as a palliative treatment, and it began its routine use in 2006, after the ESOPE study (5) and the approval of the medical grade electroporator, the Cliniporator (Igea, Carpi, Italy). Its main indications are cutaneous and subcutaneous tumors of any histology that are not candidates for other treatment modalities. Recently, it can be performed as the first approach upon patient's request, or for the treatment of internal, deep seated tumors (50). The United States and the rest of the world followed with the development of other generators. In Latin America, its use began in Argentina in 2020 after the approval of another medical grade electroporator, the OncoPore (BIOTEX SRL, Buenos Aires, Argentina). Besides the indications established in the Updated Standard Operating Procedures for ECT published by Gehl et al., intense research is undergoing to extend ECT applications to other organs, such as the liver (12), the brain (89), the pancreas (12), and the bones (12). An endoscopic electrode, the EndoVe, was developed for the treatment of colorectal cancer (90) and the esophagus (91). ECT is nowadays a valuable asset for the oncologist. It can be used alone or in combination with other therapies, and provides a new treatment option when others have failed or are not feasible (30).

The authors' personal experience comprises more than 4,000 cases, treated in various animal species, in more than 10 years of practice. That experience also includes the treatment of human patients in the clinical setting, allowing us to be aware of the relevant differences that the treatments have. It also relies on the organization of the Latin American workshops on ECT, the delivery of 9 courses to more than 150 veterinarians from Latin America and Spain, as well as the service to many users through different online platforms to answer their questions (https://vetoncologia.com/ect).

This guide provides updated practical and useful information to the veterinarians. Here we brought detailed information about important aspects that should be considered for treating the wide spectrum of patients that encompass veterinary medicine.

## Data Availability Statement

The raw data supporting the conclusions of this article will be made available by the authors, without undue reservation.

## Ethics Statement

All regulations from the Consejo Profesional de Médicos Veterinarios (Argentina) were followed. This work was approved by the IACUC of the School of Veterinary Sciences, University of Buenos Aires, Argentina. Protocol Number: 2018/31. Written informed consent was obtained from the owners for the participation of their animals in this study.

## Author Contributions

All authors equally contributed to the design, writing, proofreading of the manuscript, and approved the submitted version.

## Conflict of Interest

The authors declare that the research was conducted in the absence of any commercial or financial relationships that could be construed as a potential conflict of interest.

## Publisher's Note

All claims expressed in this article are solely those of the authors and do not necessarily represent those of their affiliated organizations, or those of the publisher, the editors and the reviewers. Any product that may be evaluated in this article, or claim that may be made by its manufacturer, is not guaranteed or endorsed by the publisher.
